# Directed Energy Transfer from Monolayer WS_2_ to Near-Infrared Emitting PbS–CdS Quantum Dots

**DOI:** 10.1021/acsnano.0c05818

**Published:** 2020-10-20

**Authors:** Arelo O. A. Tanoh, Nicolas Gauriot, Géraud Delport, James Xiao, Raj Pandya, Jooyoung Sung, Jesse Allardice, Zhaojun Li, Cyan A. Williams, Alan Baldwin, Samuel D. Stranks, Akshay Rao

**Affiliations:** † Cavendish Laboratory, Cambridge, JJ Thomson Avenue, Cambridge CB3 0HE, United Kingdom; ‡ Cambridge Graphene Centre, University of Cambridge, 9 JJ Thomson Avenue, Cambridge CB3 0FA, United Kingdom; § Department of Chemistry, University of Cambridge, Lensfield Road, Cambridge CB2 1EW, United Kingdom; ∥ Department of Chemical Engineering & Biotechnology, University of Cambridge, Philippa Fawcett Drive, Cambridge CB3 0AS, United Kingdom

**Keywords:** two-dimensional, transition metal dichalcogenide, zero-dimensional, quantum dot, tungsten disulfide, lead sulfide−cadmium sulfide, energy transfer

## Abstract

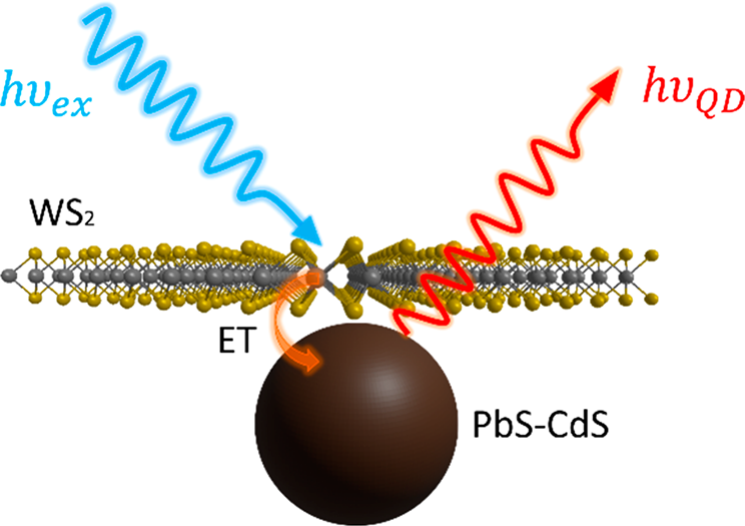

Heterostructures of two-dimensional (2D) transition metal dichalcogenides (TMDs) and inorganic semiconducting zero-dimensional (0D) quantum dots (QDs) offer useful charge and energy transfer pathways, which could form the basis of future optoelectronic devices. To date, most have focused on charge transfer and energy transfer from QDs to TMDs, that is, from 0D to 2D. Here, we present a study of the energy transfer process from a 2D to 0D material, specifically exploring energy transfer from monolayer tungsten disulfide (WS_2_) to near-infrared emitting lead sulfide–cadmium sulfide (PbS–CdS) QDs. The high absorption cross section of WS_2_ in the visible region combined with the potentially high photoluminescence (PL) efficiency of PbS QD systems makes this an interesting donor–acceptor system that can effectively use the WS_2_ as an antenna and the QD as a tunable emitter, in this case, downshifting the emission energy over hundreds of millielectron volts. We study the energy transfer process using photoluminescence excitation and PL microscopy and show that 58% of the QD PL arises due to energy transfer from the WS_2_. Time-resolved photoluminescence microscopy studies show that the energy transfer process is faster than the intrinsic PL quenching by trap states in the WS_2_, thus allowing for efficient energy transfer. Our results establish that QDs could be used as tunable and high PL efficiency emitters to modify the emission properties of TMDs. Such TMD-QD heterostructures could have applications in light-emitting technologies or artificial light-harvesting systems or be used to read out the state of TMD devices optically in various logic and computing applications.

Monolayer transition metal dichalcogenides (TMDs), which are derived from their layered bulk crystals *via* dry mechanical cleavage^1^ or liquid-phase exfoliation,^2,3^ have attracted a great deal of research interest due to their distinctive optical, electronic, and catalytic properties.^4−6^ Monolayer TMDs can also be obtained *via* epitaxial growth methods, in particular, chemical vapor deposition (CVD),^7,8^ which is an area of ongoing research. A number of monolayer TMDs such as tungsten disulfide (WS_2_) have a direct optical gap.^5^ This property compounded with high absorption coefficients, high carrier mobilities,^5^ and potentially high photoluminescence quantum efficiency^9−11^ (PLQE) promises great potential for their application in optoelectronic devices, namely, photodetectors, light-emitting diodes (LEDs), and photovoltaics (PV).^12^ The reduced dielectric screening in the monolayer limit compared to that of their bulk counterparts gives rise to tightly bound electron–hole pairs (*i.e.*, excitons) with binding energies on the order of hundreds of millielectronvolts at room temperature.^13,14^ As a consequence, monolayer TMDs provide a convenient medium to study diverse excitonic species that arise *via* exciton–exciton or exciton–charge interaction.^13,15−17^ Alternatively, these tightly bound excitons can be funneled to other fluorescent media, where they recombine radiatively at lower energy, thus tuning the emission properties of TMD excitons. Nanocrystal quantum dots (QDs), for example, provide a convenient, color-tunable high PLQE emission medium^18,19^ to which transferred 2D TMD excitons might be funneled.

The exciton funneling, that is, a nonradiative energy transfer (ET) process, can occur *via* two main mechanisms, namely, Förster resonance energy transfer^20^ (FRET) and Dexter energy transfer (DET).^21^ FRET is a long-range process (∼1–11 nm)^20^ that occurs *via* dipole–dipole coupling, where the electromagnetic near-field of an oscillating transition dipole in the donor induces a transition dipole in the acceptor. Consequently, FRET between donor and acceptor systems is dependent on their physical separation and, to a large extent, the overlap of emission and absorption spectra.^20−22^ On the other hand, DET involves direct simultaneous tunneling of electron–hole pairs from the donor to the acceptor due to donor–acceptor charge orbital overlap. As such, DET is strongly distance-dependent and requires extremely close proximity between donor and acceptor molecules (≤1 nm).^21,23^

A considerable amount of research into 2D-QD heterostructures has focused on interfacial charge transfer (CT) between QDs and monolayer TMDs for applications in photodetectors^24−31^ and phototransistors.^32,33^ To date, studies on the energy transfer in 2D-QD heterostructures for light-harvesting and light-sensing applications have mainly focused on 0D→2D exciton transfer where monolayer TMDs or graphene are used as efficient exciton sinks to which optically or electrically generated excitons from QD emitters are nonradiatively transferred.^22,30,34−39^

Here, we demonstrate efficient ET from 2D TMDs to 0D QDs. We present a down-shifting heterostructure system, where monolayer tungsten disulfide acts as an antenna from which optically generated excitons are funneled to lower-energy lead sulfide–cadmium sulfide (PbS–CdS) near-infrared (NIR) QD emitters. Photoluminescence excitation (PLE) studies confirm 2D→0D ET. Probing the underlying photophysics *via* time-resolved optical microscopy reveals a fast, nonradiative ET process that out-competes intrinsic exciton trapping in monolayer WS_2_. These results establish ET from 2D TMDs to 0D QDs as an efficient means to control excitonic behavior, allowing for tuning of emission energies and construction of artificial light-harvesting systems.

## Results and Discussion

**Figure 1:**
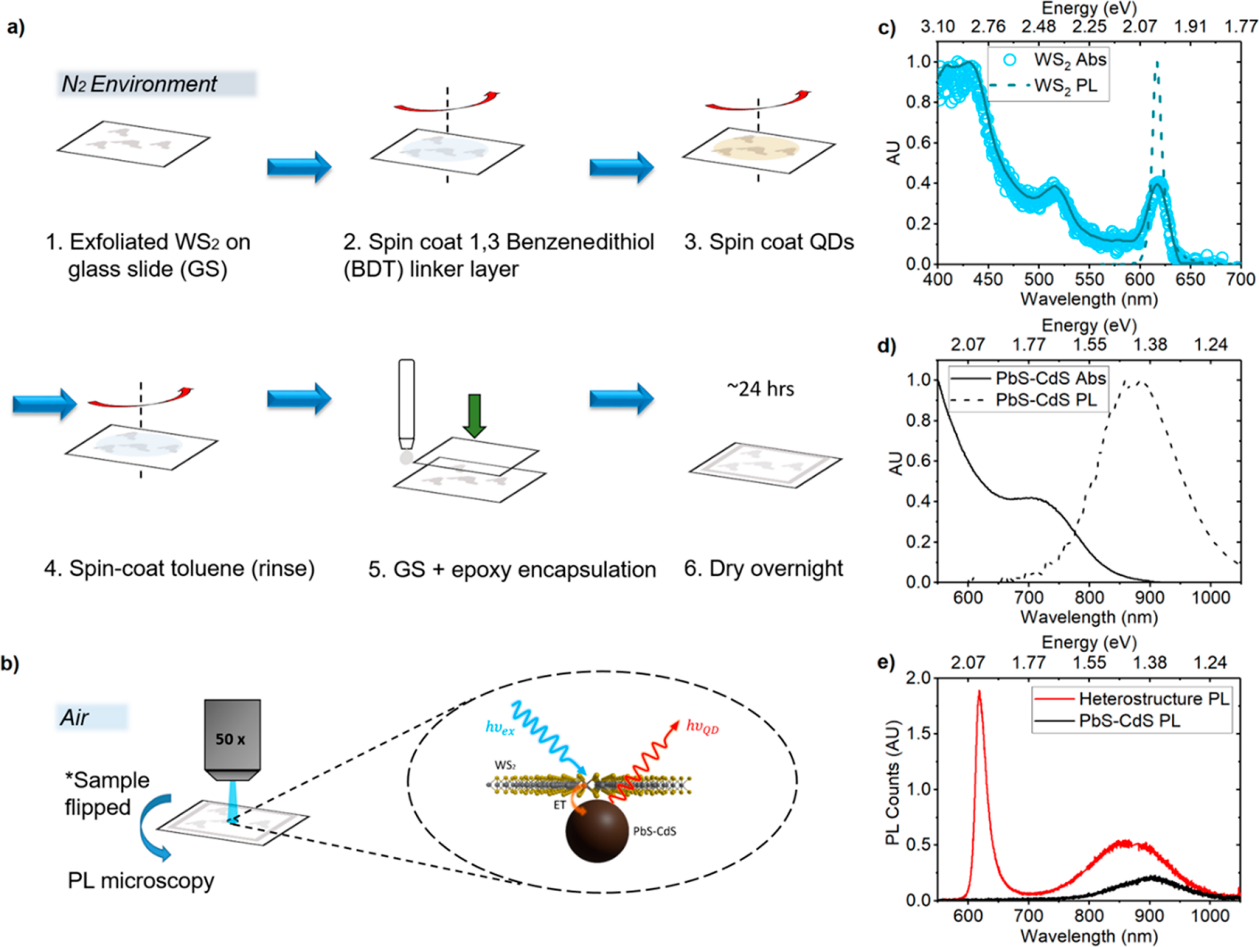
(a) Cartoon illustrating heterostructure sample fabrication process (1–6) and (b) initial PL characterization *via* 50× objective. (c) Monolayer WS_2_ normalized absorption (light blue circles with solid dark blue line as a guide for the eye) and PL (dashed dark blue line). (d) Colloidal PbS–CdS normalized absorption (black solid line) and PL (black dashed line) spectra. (e) PL spectra of WS_2_–PbS–CdS 2D-QD heterostructure (red) and PbS–CdS film on bare glass substrate (black) measured with 514.5 nm continuous wave laser at 80.2 W/cm^2^.

Figure 1a (1–6) shows the sample fabrication process from the initial exfoliated monolayers to the heterostructure. Following monolayer WS_2_ exfoliation, a single QD layer was deposited on the sample surface using a conventional layer-by-layer method:^40,41^ A linker layer of 1,3-benzenedithiol (BDT) was first deposited *via* spin-coating to ensure strong adhesion of QDs on the sample surface; a low concentration (0.5 mg mL^–1^) of oleic acid (OA)-capped PbS–CdS QDs was then spun onto the sample; and excess nanocrystal and ligand material was rinsed off by spin-coating toluene, leaving a single layer of QD film. Sample preparation is detailed further in the Experimental Methods section. Figure 1b illustrates the process of exciting the 2D material with high-energy visible photons, forming excitons that funnel to the QDs where they recombine and emit lower-energy NIR photons. Figure 1c shows the absorption and PL spectra of a WS_2_ monolayer. The absorption spectrum of the WS_2_ monolayer (light blue circles) clearly reveals “A”, “B”, and “C” excitonic peaks positioned at 2.0 eV (617 nm), 2.4 eV (512 nm), and 2.88 eV (430 nm), respectively. The PL spectrum (dark blue dashed line) is well overlapped with the A exciton band. The absorption and PL spectra of the QDs in the colloidal suspension are plotted in Figure 1d. The colloidal PbS–CdS absorption spectrum (solid black line) reveals an absorption peak at 1.76 eV (704 nm), whereas the PL spectrum (black dotted line) exhibits the red-shifted peak position at 1.38 eV (900 nm). Interestingly and importantly, the WS_2_ PL lies within the PbS–CdS absorption spectrum, which is a key requirement for efficient FRET. Consequently, we chose PbS–CdS QDs and a WS_2_ monolayer as an efficient energy transfer pair.

An additional factor considered was the absorption cross sections of the constituent donor and acceptor materials. The TMD monolayer’s role as an optical antenna and exciton generation medium requires that it has a higher absorption cross section compared to the nanocrystal emitter. Whereas the absorption cross sections of monolayer tungsten disulfide and other TMDs in the visible region are not very well documented, the absorption coefficient, σ_gs_, of few-layer (1–3 monolayers) MoS_2_ obtained from a study on nonlinear optical performance of MoS_2_ films by Zhang *et al.*(2) gives a value of σ_gs_ = 4.7 × 10^–15^ cm^2^ for 515 nm pulsed excitation. We estimate the absorption cross section for a MoS_2_ monolayer simply by dividing σ_gs_ = 4.7 × 10^–15^ cm^2^ by the maximum number of layers (*n* = 3) in the sample quoted to give σ_gs_ ≈ 1.6 × 10^–15^ cm^2^ at 515 nm. We note that the absorption of monolayer WS_2_ is similar in magnitude to that of MoS_2_ at 515 nm^42^ and hence estimate that their absorption cross sections are comparable at 515 nm. Moreover, considering the transition from indirect to direct optical gap from few-layer to monolayer TMD, the actual value of absorption cross section should exceed this estimation. Following Cademartiri *et al.*,^43^ we compute the absorption cross section of a single QD *via*eq 1 using the molar extinction coefficient, ε_A_ (M^–1^ cm^–1^) estimated in Supporting Information (SI) section 3.2. Units are provided in square brackets for clarity.
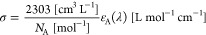
1where *N*_A_ is the Avogadro number. This yields a value of σ ≈ 8.74 × 10^–17^ cm^2^ at 515 nm. Given the estimations made and the shape of the monolayer WS_2_ absorption spectrum (Figure 1c), we consider that the WS_2_ absorption cross section exceeds that of the QDs by a large factor in the ∼400–650 nm range.

As shown in the Figure 1b, steady-state PL microscopy was performed with the sample placed upside down to directly excite the monolayer WS_2_ first *via* the thin glass slide, avoiding shadowing by the QDs. Directly exciting the monolayer ensures efficient generation and funneling of TMD excitons to the QDs, as illustrated in Figure 1b (inset). This results in considerable QD PL enhancement in the heterostructure, as subsequently discussed in detail for Figure 1e. Exciting the QDs directly would otherwise cause suboptimal exciton generation and funneling from the monolayer TMD due to absorption of a proportion of incoming photons by the shadowing QDs, amounting to less prominent QD PL enhancement. The steady-state confocal PL spectra of the QD film on the bare substrate (black) and the heterostructure (red) are plotted in Figure 1e. Although the QD film on the bare substrate shows a broad Gaussian PL peak in the NIR region centered at 1.38 eV (900 nm), the heterostructure exhibits two distinctive PL peaks, that is, the narrow WS_2_ PL peak in the visible region centered at 2.0 eV (∼619 nm) and a broad QD PL peak in the NIR region at 1.42 eV (870 nm). We note that the QD PL spectrum of the heterostructure is blue-shifted by 30 nm and enhanced by a factor of 2.6. The observed blue shift in the heterostructure’s QD PL on the WS_2_ monolayer compared to that on the bare substrate may be attributed to the following possibilities: (i) a difference in dielectric environment between the surfaces; (ii) a difference in QD aggregation concentration of the QD film between the surfaces; or (iii) a combination of both factors. We also note that it is possible that the PL yield of the QDs on the WS_2_ monolayer is higher than those on the bare substrate as a result of the aforementioned factors. Whereas ascertaining the nature of the heterostructure’s surface morphology and dielectric properties could offer additional explanation toward the observed changes in QD emission properties between the bare substrate and TMD monolayer surface, the scope of this work is confined to investigating the possibility of ET of WS_2_ excitons to the QDs as evidenced by the QD PL enhancement on the monolayer surface.^21^ Hence, we seek to verify the notion of 2D→ QD ET *via* further optical characterization studies.

**Figure 2:**
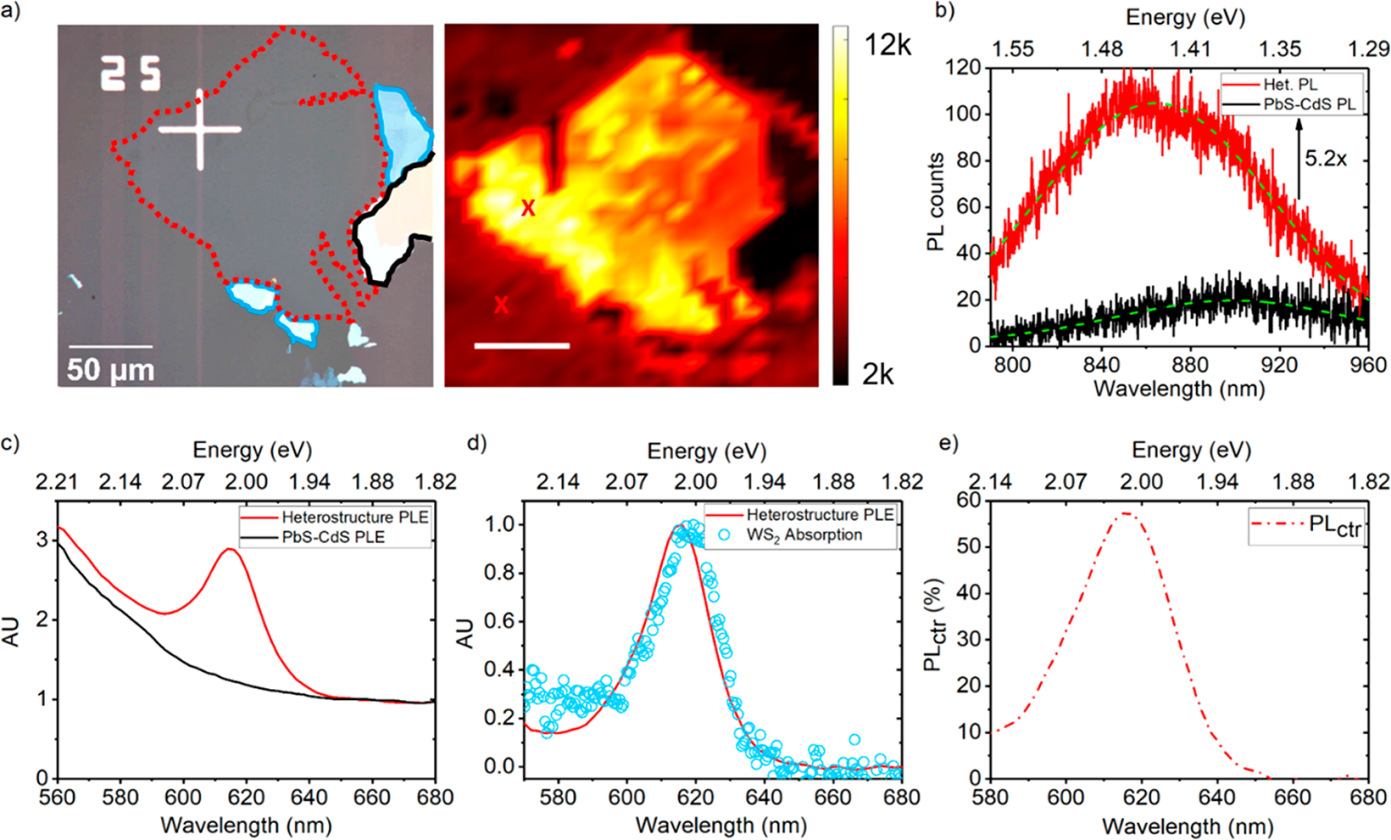
(a) Optical micrograph of a WS_2_ flake (left) showing monolayer (red dotted outline), multilayers (blue outline), and bulk crystal (black outline) with corresponding confocal NIR PL map of QD emission from the heterostructure (right) measured with a 514.5 nm continuous wave laser at 80.2 W/cm^2^. Right-hand side scale bar represents 50 μm. (b) QD PL spectra from heterostructure (red) and bare substrate (black) taken from points marked “*x*” in (a), right-hand side. Green dashed lines represent single Gaussian peak fits. (c) Normalized PLE spectra of heterostructure (red) and QD (control) obtained *via* scanning wavelengths about the WS_2_ “A” exciton (616 nm) and detecting QD PL (900 nm). PLE spectra normalized by the average signal between 670 and 700 nm. (d) Normalized “subtract” (red) signal derived *via* subtraction of QD PLE signal from heterostructure PLE signal in (b) and overlapped with typical WS_2_ absorption spectrum (blue circles). (e) Estimated contribution to QD PL (PL_ctr_) by the WS_2_ monolayer as a function of excitation wavelength with peak value of 58% at 616 nm (∼2.0 eV).

Figure 2a shows the optical micrograph (left) of a WS_2_ flake and confocal NIR (QD) PL map (right) from the same region obtained upon excitation at 514.5 nm. The color bar represents the PL integral in the 780–960 nm spectral range. Enhanced NIR PL from QDs is obtained in the vicinity of the monolayer (dashed line), whereas QD PL in the bulk flakes (solid line) is quenched. The difference in NIR PL intensity between monolayer and bulk flakes suggests that the WS_2_ monolayer serves as the ET donor, whereas the bulk quenches excitons. Figure 2b shows the QD PL spectra from the heterostructure (red) and bare substrate (black) extracted from points marked “*x*” on the QD PL map in Figure 2a, right-hand side. Lime green dashed lines are single Gaussian peak fits. The QD PL spectrum of the heterostructure is blue-shifted by 47 nm compared with the QD on the bare substrate. We also observe a QD PL enhancement of 5.2×, which we attribute to energy funneling from the directly excited WS_2_ monolayer. To delve into the possibility of ET from the WS_2_ monolayer to PbS–CdS QDs, we employ wide-field PLE microscopy. We recorded the PL intensity integrated over the NIR region (800–1000 nm), exclusively corresponding to PL from the QDs, and scanning the excitation wavelength across 560–700 nm, mainly resonant to WS_2_ at low fluence (∼0.006 μJ/cm^2^ at 620 nm). The PLE spectra shown in Figure 2c were taken on the heterostructure (red) and in an area with QDs only (black) away from the heterostructure. We note that the PLE data are normalized with respect to the mean PLE values at wavelengths off-resonant to the WS_2_ donor (670–700 nm) to account for the increase in QD emission due to resonant 2D → QD ET only, discounting the effects of other previously discussed factors that may contribute to improved QD emission on the heterostructure. The unscaled PLE data for Figure 2c are included in SI section 1. Unlike the PLE spectrum of the QD-only area (black), the PLE spectrum of the heterostructure (red) clearly reveals the signature “A” exciton peak centered at 616 nm (∼2.0 eV), indicative of a significant contribution from the WS_2_. Furthermore, as shown in Figure 2d, the resulting PLE spectrum (red line) obtained by subtracting the normalized QD PLE spectrum (Figure 2c., black) from that of heterostructure (Figure 2c, red) is almost perfectly overlapped with a typical WS_2_ absorption spectrum (blue circles). This is strong evidence that energy transfers from the WS_2_ monolayer to the QDs. In order to accurately quantify ET from the WS_2_ monolayer to the QD, we calculated the photoluminescence contribution, PL_ctr_, as a function of excitation wavelength using PLE data shown in Figure 2c. The key assumption in the derivation of PL_ctr_ is informed by Vavilov’s rule,^44^ which states that PLQE is independent of excitation wavelength. QD PLQE is hence regarded as constant. This is considered as a reasonable assumption for the wavelength range presented in the PLE data (560–680 nm). Further details on the derivation of PL_ctr_ are given in SI section 2. As shown in Figure 2e, PL_ctr_ is maximized at 616 nm with a value of 58% and decreases considerably thereafter at lower-energy excitation energy. Additionally, we carried out PLE measurement on a series of heterostructures with various 2D-QD surface attachment thiol ligands. As well as the heterostructure based on BDT reported herein, 1,4-butanedithiol and 1,6-hexanedithiol were also studied. SI section 3.1 provides a brief PLE study of the heterostructures based on the different ligands. From this, we note here that all heterostructures measured show ET from 2D to QD.

All surface attachment ligands used have lengths <1 nm and thus, in principle, lie within the range for ET *via* tunneling (*i.e.*, DET). Although orbital overlap between the monolayer TMD donor and QD acceptor is a possibility at such separation distances, their respective large oscillator strengths highly favor ET *via* FRET^21^ over DET. In SI section 3.2, we estimated the theoretical Förster radius of *R*_0_ ≈ 6.5 nm, which exceeds the lengths of the ligands used. This result emphasizes the significance of the combined oscillator strengths of the constituent heterostructure materials (*i.e.*, TMD donor and QD acceptor) over their physical separation, even at low proximity, which strongly suggests FRET as the dominant ET mechanism observed in the heterostructures measured. In addition, whereas short ligands such as BDT have previously been shown to improve CT between QDs,^45^ the CdS shell encapsulating the PbS core has been shown to suppress CT.^46^

To gain further insight into the dynamics of the ET process observed from PLE, we turn to time-resolved PL (TRPL) microscopy, where we detected changes in emission decay from WS_2_ using a 509 nm pulsed laser excitation. Excitation was filtered from the detection line with a 510 nm long-pass filter and QD emission was removed using a 700 nm short-pass filter, allowing for WS_2_ monolayer PL detection only. To distinguish bare WS_2_ from WS_2_ in the heterostructure, we refer to the former as “pristine” WS_2_.

**Figure 3:**
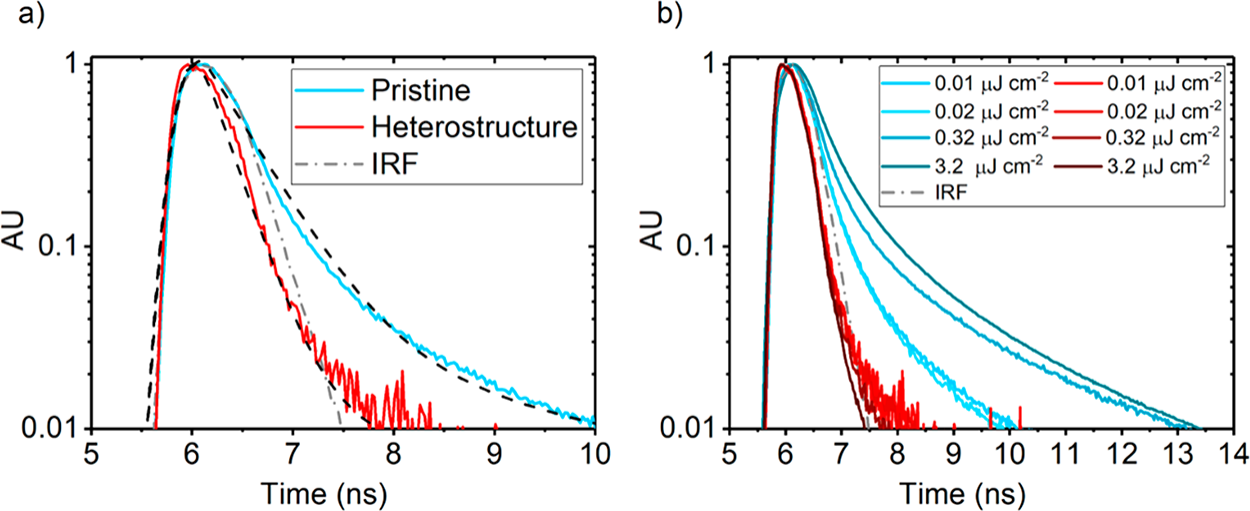
(a) Low fluence time-resolved WS_2_ PL decay signals from pristine (blue) and heterostructure (red) samples measured with 509 nm pulsed excitation at 0.01 μJ/cm^2^. Exponential decay fits are shown as dotted black lines. (b) Time-resolved WS_2_ PL decay fluence series from pristine (blue) and heterostructure (red) samples. Pristine WS_2_ PL decay signals show general increase in lifetime as a function of pump fluence due to exciton trapping. All WS_2_ PL in the heterostructure signal quenched below IRF (gray dash-dotted line) due to fast exciton transfer.

Figure 3a shows the normalized TRPL decay signals of the pristine monolayer and heterostructure under low fluence excitation (0.01 μJ cm^–2^). The transient PL profile of pristine WS_2_ shows a biexponential decay profile consisting of fast and slow components. On the other hand, we observe that the fast component of the heterostructure’s PL profile is quenched below the detector’s initial response function (IRF). The two PL decay components observed in the pristine monolayer can be attributed to direct band-edge to ground-state exciton transitions and exciton trapping, respectively.^11^ In contrast, the much faster PL kinetics observed in the heterostructure suggests an additional efficient fast decaying process present in this system. In fact, this quenching observed in the heterostructure is in accordance with what is expected of the PL dynamics of the donor in a nonradiative ET system. Figure 3b shows an excitation fluence series performed on both pristine and heterostructure samples. The pristine case shows a general increase in PL lifetime with fluence, which is indicative of “trap” or “defect” state filling. This trap-limited behavior has also been observed in WS_2_ and MoS_2_ monolayers treated with bis(trifluoromethane)sulfonimide.^11,47^ The apparent increase in the fast component of the PL lifetime with fluence is due to trapping and detrapping of excitons to the band-edge prior to recombination to the ground state. The long-lived component is due to radiative transitions from the trap to ground state.^47^ Increasing the excitation fluence would lead to saturation of trap states, forbidding further trapping and promoting dominant band-edge to ground-state recombination. The fluence series presented in Figure 3b, however, lies below trap-state saturation. This is given by the increasing fast PL component lifetimes as a function of fluence. Trap-state saturation would otherwise be characterized by a constant fast PL component with increasing excitation fluence. Further increases in fluence would lead to an eventual reduction of the fast PL component lifetime, signaling the onset of exciton–exciton annihilation. Interestingly, in the heterostructure case, the fast PL components are quenched below the IRF throughout the series. This outcome suggests that ET rate outcompetes the intrinsic exciton trapping rate in monolayer WS_2_, which occurs on a time scale of a few picoseconds.^48,47^ We therefore predict that the 2D → QD ET rate occurs on a faster or similar time-scale.

**Figure 4:**
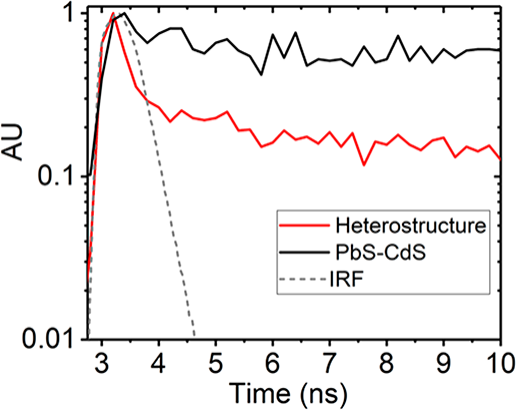
QD TRPL decay spectra of heterostructure (red) and bare substrate (black) measured with 509 nm pulsed excitation at 0.5 MHz. The early time signal in heterostructure PL decay convoluted with IRF confirms that ET phenomenon faster than resolution of TCSPC setup available for this study.

The observation of a concomitant growth in QD PL lifetime with WS_2_ PL quenching would provide further confirmation of ET. However, as recently discussed, it is likely that the ET process occurs on a time scale faster than intrinsic trapping in the monolayer TMD (*i.e.*, a few picoseconds), too fast to be detected by time-correlated single-photon counting (TCSPC), as employed in this study, and perhaps even too fast for streak camera measurements. As further confirmation of this hypothesis, Figure 4 shows the normalized TRPL decay signals for a heterostructure (red) prepared on Spectrosil compared with the QDs on the bare substrate (black). Excitation was provided using the 509 nm pulsed laser at a 0.5 MHz repetition rate and a 200 ps resolution. The excitation signal was filtered out using a 510 nm long-pass filter, and QD emission was isolated with an 800 nm long-pass filter, removing any signal from the underlying WS_2_. The heterostructure decay clearly shows the IRF component convoluted with the long-lived QD PL decay at an early time. This indicates the occurrence of a phenomenon much faster than the sensitivity of the setup. Therefore, the expected increase in QD lifetime due to ET from the underlying WS_2_ occurs at a much earlier time than what is detectable by the TRPL setup available to us.

**Figure 5:**
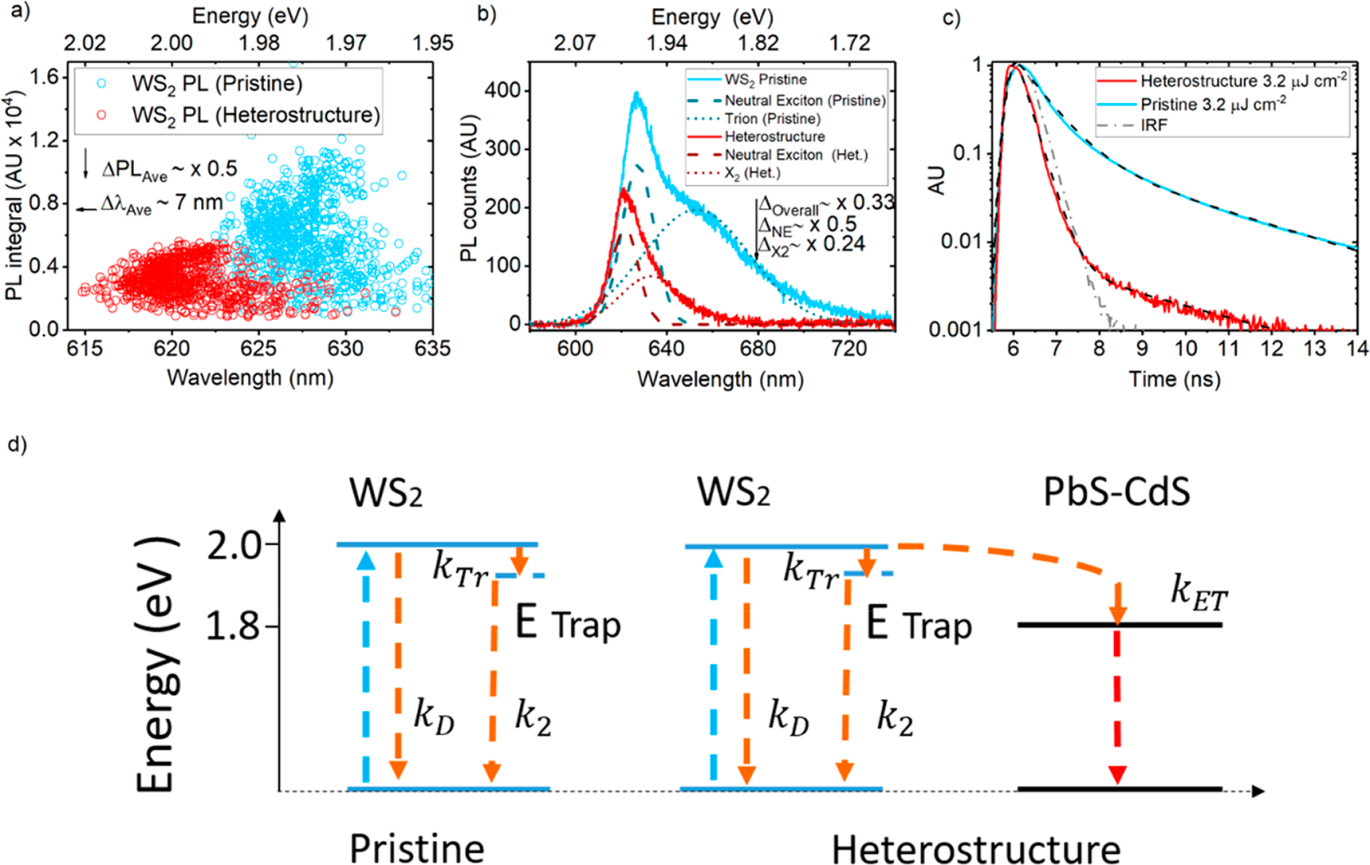
(a) Scatter plots of monolayer WS_2_ (visible) PL integrals and the corresponding PL peak wavelengths extracted from spatial PL maps of the sample in pristine (blue) and heterostructure (red) form. PL measured with 514 nm continuous wave laser excitation at 80.2 W cm^–2^ intensity. (b) WS_2_ PL spectra of an example of the monolayer in pristine (blue) and heterostructure (red) cases. Spectra are deconvoluted with Gaussian peaks which represent the neutral exciton (dashed lines) and a lower-energy species X_2_ (dotted lines). (c) TRPL decay spectra of pristine (blue) and heterostructure (red), measured with 509 nm excitation at 63.4 W cm^–2^ intensity. Black dashed lines represent decay fits. IRF given by gray dot-dash line. (d) Energy level diagram illustrating radiative exciton pathways in pristine WS_2_ (left-hand side) and in the heterostructure. Blue arrows represent initial excitation; orange arrows represent WS_2_ excitons, and red arrows represent down-shifted excitons that recombine at lower energy in the PbS–CdS QD.

Steady-state PL measurements provide information on the spectral changes that occur in the WS_2_ monolayer PL from pristine to the heterostructure case. Also, comparing steady-state PL with TRPL data at similar excitation intensity provides a better understanding of exciton recombination pathways in the heterostructure. Figure 5a shows scatter plots of monolayer WS_2_ (visible) PL integrals and the corresponding PL peak wavelengths extracted from spatial PL maps of the sample in pristine (blue) and heterostructure (red) form. These data were collected from a 64 μm × 48 μm rectangular region within the monolayer shown in Figure 2a, left-hand side. As far as practicable, PL measurements were taken in the same region before and after QD deposition. For further clarity, the WS_2_ PL collection region is shown in SI section 4. Maps were measured with 514 nm continuous wave (CW) laser excitation at 80.2 W cm^–2^ intensity for a good signal-to-noise ratio. It is known that different types of excitons exist in atomically thin nanomaterials (*i.e.*, WS_2_ monolayer). Accordingly, it is of importance to understand how different types of excitons behave and contribute differently when ET occurs. We begin with analyzing steady-state PL spectra as it gives an indication of the types of excitons present. Figure 5b shows the PL spectra of an exemplary point on the monolayer in pristine (blue) and heterostructure (red) form. The spectra were deconvoluted with Gaussian peaks, which represent the neutral exciton (NE) and lower-energy species (X_2_) such as trions, which are characterized by broad low-energy features in monolayer TMD spectra.^11^ X_2_ may also arise from eventual radiative recombination of neutral excitons trapped in subgap states. Upon recombination to the ground state, these excitons can bind with electrons to form trions, which is known to occur in *n-*type TMDs such as WS_2_.^11,49^Figure 5c shows the fitted TRPL of pristine (blue) and heterostructure (red) cases at high excitation intensity (3.2 μJ cm^–2^ → 63.4 W cm^–2^). Figure 5d shows the proposed radiative exciton recombination pathways resulting from the high-intensity PL/TRPL comparison. Table 1 shows the fitted PL lifetimes (τ) of pristine and heterostructure samples at low and high intensity excitation and ET efficiencies. ET efficiencies were computed *via*eq 2. SI section 5 provides the full derivation of eq 2. Heterostructure lifetimes are denoted by an apostrophe. Given that the fast component of the heterostructure’s WS_2_ PL lifetime (τ_1_′) is limited by the IRF, the fitted values presented in Table 1 represent an upper bound.
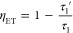
2

**Table 1:** Fitted PL Lifetimes of Pristine and Heterostructure Samples and Resulting Estimates for ET Efficiencies^a^

intensity	pristine τ_1_	heterostructure τ_1_′	pristine τ_2_	heterostructure τ_2_′	η_ET_
0.21 W cm^–2^	0.456 ns	0.26 ns	3.63 ns	3.64 ns	42%
***63.4* W cm^–2^**	*0.62 ns*	*0.26 ns*	*2.95 ns*	*2.9 ns*	*58%*

a Fast components of WS_2_ PL decay in the heterostructure, τ_1_′, and transfer efficiencies, η_E__T_, represent upper and lower bound values, respectively, due to limitations in instrument sensitivity. High-intensity excitation values used for comparison with steady-state PL are italicized.

Statistical analysis of the scatter data in Figure 5a reveals an average PL quenching, ΔPL_AV__E_ = 50%, and spectral blue shift, Δλ_AVE_ = 7 nm, from the pristine to the heterostructure case. The spectra in Figure 5b show that the NE component quenches by 50%, whereas X_2_ quenches by 76%. An overall quenching of 67% was computed from the raw spectra. The large X_2_ quenching helps to explain the spectral narrowing in the red signal and the general blue shift in Figure 5a. Interestingly, the difference in quenching between the NE and X_2_ species leaves 26% of quenched excitons unaccounted for. This implies an additional exciton recombination pathway. As X_2_ may arise from slow exciton recombination from trap states, the excess quenching of X_2_ excitons could be explained as nonradiative trap→QD transfer. Table 1, however, reveals that the slow decay component (τ_2_) associated with the trap→ground-state transition remains practically unchanged between the pristine and heterostructure case for a given excitation intensity (*i.e.*, τ_2_ ∼ τ_2_′). The WS_2_ trap state to QD exciton transfer requires that τ_2_′ < τ_2_ and therefore negates this possibility. This suggests that the excess quenched excitons may dissipate *via* some other nonradiative pathway.

On the other hand, Table 1 shows that the fast component of the biexponential decay associated with neutral exciton recombination^11^ is quenched by 58% from τ_1_ ∼ 0.62 ns in the pristine monolayer to τ_1_′ ∼ 0.26 ns in the heterostructure case. This lies in close agreement with the 50% NE quenching estimated in steady-state PL. The strong fast PL decay lifetime quenching shows that ET occurs *via* neutral excitons transitioning from the WS_2_ band-edge to the QD acceptor, whereas intrinsic exciton trapping in the donor and nonradiative losses compete with this process. This justifies the use of fast decay components (τ_1_, τ_1_′) to compute the lower bound ET efficiencies shown in Table 1*via*eq 2. As previously highlighted, exciton trapping and detrapping in the donor gives rise to increasing τ_1_ as a function of fluence, which manifests as an apparent increase in η_ET_ as a function of fluence. Although nonradiative pathways are yet to be uncovered, passivating trap states to improve donor PLQE should lead to more efficient ET from the WS_2_ donor band-edge to the QD acceptor.

Figure 5d provides a clear illustration of radiative exciton pathways in pristine (left-hand side) and heterostructure (right-hand side) cases, which is derived from the PL/TRPL comparison in Figure 5b,c and supported by the TRPL fluence series in Figure 3b. In pristine WS_2_, upon excitation from the ground state, a proportion of excitons instantaneously transition from the band-edge to trap states on the order of a few picoseconds^47^ at the trapping rate, *k*_TR_, whereas others recombine radiatively from the band-edge to ground state at the rate of *k*_D_. Those excitons that are trapped in subgap states radiatively recombine to the ground state over long periods on the order of nanoseconds^47^ at the rate *k*_2_. In the heterostructure, excitons preferentially transfer from the WS_2_ band-edge to the QD at the rate *k*_ET_, such that *k*_ET_ > *k*_TR_, thus quenching the fast component τ_1_ lifetime below the IRF. This also explains the sizable quenching of X_2_ in the steady-state PL spectra as there are fewer excitons being trapped in the presence of an acceptor QD. Band-edge excitons that are not trapped, transferred, or lost *via* some other nonradiative process recombine radiatively to the ground state at *k*_D_ over tens to hundreds of picoseconds,^48^ which is below the instrument response. The remaining emission from direct band-edge recombination, as shown in Figure 5b, strongly suggests that the 2D→QD transfer pathway becomes saturated. As with trap states, the QD band-edge can become saturated, forbidding further incoming excitons, which may return to the WS_2_ band-edge and radiatively recombine or dissipate *via* a nonradiative process as suggested by the “lost” quenched excitons identified from Figure 5b.

To summarize the results from optical measurements presented, PLE studies confirm ET from monolayer 2D WS_2_ to 0D QDs. Further PLE on heterostructures with differing surface attachment thiol ligands show ET. Whereas all ligand lengths used lie within tunneling distances favorable for DET (<1 nm), the large oscillator strengths of the 2D TMD donor and QD acceptor favor FRET, as given by the large theoretical Förster radius computed. The CdS shell surrounding the PbS core in the QDs provides an additional tunneling barrier, thus supporting FRET as the dominant ET process observed. TRPL studies further confirm nonradiative ET by virtue of strong quenching of donor WS_2_ PL in the presence of the acceptor QDs. TRPL studies also strongly indicate that this transfer process is faster than intrinsic early time trapping of excitons in the WS_2_ monolayer, which would otherwise lead to radiative or nonradiative exciton recombination *via* trap states in the pristine monolayer. Comparing high excitation intensity PL and TRPL measurements provides a clearer understanding of radiative recombination pathways for excitons in the TMD QD heterostructure. The comparison implies that intrinsic exciton trapping in the TMD monolayer and a nonradiative process compete with ET from 2D to QD. Further analysis also suggests that the exciton transfer channel can become saturated at high excitation intensities.

## Conclusions

In conclusion, we have demonstrated the ability to transfer excitons from monolayer WS_2_ to NIR PbS–CdS QD emitters. PLE studies provide confirmation of ET, with 58% of QD PL donated by monolayer WS_2_. The large oscillator strengths of the donor TMD and acceptor QD lead to a large Förster radius, suggesting FRET as the dominant ET mechanism. TRPL studies reveal that the ET process is faster than intrinsic exciton trapping in monolayer WS_2_. A comparative study between high excitation steady-state PL and TRPL confirms exciton transfer from the WS_2_ band-edge to the PbS–CdS band-edge, whereas intrinsic exciton trapping in the donor and other nonradiative channels act as competing pathways. Residual emission from the donor in the heterostructure suggests that the ET pathway can be saturated at high excitation intensities. Future studies of such heterostructures could provide a clearer understanding of nonradiative loss mechanisms *via* more sensitive methods such as femtosecond transient absorption and high-resolution TRPL. Trap-state passivation *via* monolayer TMD surface treatments can be used to drastically reduce exciton trapping rates, not only enhancing ET but also isolating nonradiative loss pathways so that they can be better understood. The TMD/QD heterostructures demonstrated here combine the high absorption cross section or electrical injection and transport properties of monolayer TMDs, with the high-quality and highly tunable optical properties of QDs. The ability to tune emission properties of monolayer TMDs using high PLQE QD emitters has potential device applications in areas such as in light-emitting technologies, namely, displays, solid-state lighting, and lasers,^19,22^ as well as artificial light-harvesting systems. Such structures could also be used to read out the state of TMD devices optically in various logic and computing applications.

## Expermental Methods

### Sample Preparation

#### Monolayer Preparation

Thin 22 mm × 22 mm glass cover slides with a thickness of 170 μm were solvent processed *via* sonication in acetone and isopropyl alcohol (IPA) for 15 min, dried with a nitrogen (N_2_) gun, and treated in oxygen (O_2_) plasma to remove adsorbants. Large-area WS_2_ monolayers were prepared *via* gold-mediated exfoliation.^50^ The bulk crystal was purchased from 2D Semiconductors and exfoliated manually onto low adhesion clean-room tape prior to depositing a thin gold layer (∼100–150 nm) *via* thermal evaporation under vacuum conditions. Once gold was evaporated, thermal release tape was adhered atop the gold-coated WS_2_ and peeled, leaving exfoliated WS_2_ on top of a layer of gold attached to the thermal release tape. With the WS_2_ exfoliate facing downward, the thermal tape was affixed to the target substrate and heated on a hot plate up to 125 °C. Once the thermal tape was peeled, leaving the WS_2_ exfoliate sandwiched between the substrate and gold, the excess gold was removed by gently swirling the sample immersed in potassium iodide (KI_2_) and iodine (I_2_) standard gold for 6 min. Finally, the sample was rinsed in deionized water and then sonicated in acetone for 10 min and rinsed in IPA for 5 min. Samples were dried with a nitrogen gun. Monolayers were identified using an optical contrast method.^51^

#### Pbs–CdS QD Preparation

All chemicals were purchased from Sigma-Aldrich or Romil and were used as received. The synthesis of PbS QDs was carried out following modified versions of the method of Hines and Scholes.^52^

Lead oxide (0.625 g, 99.999%), oleic acid (OA, 2 mL, 90%), and 1-octadecene (ODE, 25 mL, 90%) were placed in a three-necked round-bottomed flask and degassed under vacuum at 110 °C for 2 h with stirring, forming a colorless solution. Subsequently, the flask was put under nitrogen flow and heated to 80 °C. In a nitrogen glovebox, a syringe was prepared containing ODE (13.9 mL) and bis(trimethylsilyl)sulfide (296 μL, 95%). The syringe containing the sulfur precursor was rapidly injected into the reaction flask, which was allowed to cool. Upon cooling to 60 °C, the reaction mixture was transferred to an argon glovebox. The synthesized nanocrystals were purified four times by precipitation with ethanol/1-butanol and acetone, centrifugation (10000*g*), and resuspension in hexane/toluene. The purified QDs were redispersed in toluene for storage in an argon glovebox.

Cation exchange of PbS QDs was performed following a modified method of Neo *et al.*(53) A typical procedure was as follows:

Cadmium oxide (1.03 g, 99.999%), OA (6.35 mL), and ODE (25 mL) were placed in a three-necked round-bottomed flask and degassed under vacuum for 110 °C. The vessel was switched to nitrogen and heated to 230 °C for 2 h, resulting in the formation of a colorless solution of cadmium oleate. The solution was cooled and degassed under vacuum for 15 min. The flask was switched to nitrogen, and the solution was transferred to a nitrogen glovebox for storage. The cadmium oleate precipitated at room temperature and was heated to 100 °C before use.

Cation exchange was performed with the addition of cadmium oleate solution to PbS nanocrystals. A typical reaction is as follows. In a nitrogen glovebox, a suspension of PbS nanocrystals in toluene (50 mg, 50 mg mL^–1^) was heated to 100 °C. Cadmium oleate in ODE (0.35 mL, 0.26 M) was added to the nanocrystal suspension and maintained at 100 °C. The reaction was quenched after 1 min with the addition of anhydrous acetone. The cation-exchanged nanocrystals were twice precipitated, centrifuged, and resuspended with acetone and toluene.

#### Heterostructure Preparation

Heterostructures were prepared using the following steps: In a nitrogen glovebox, the monolayers on the substrate were spin-coated at 1000 rpm for 50 s with 200 μL of 20 mM 1,3-benzenedithiol dissolved in acetonitrile, forming a linker layer; 200 μL of 0.5 mg/mL PbS–CdS QDs, with OA surface attachment ligands suspended in toluene were deposited *via* spin-coating at 500 rpm for 60 s; excess material was rinsed off by spin-coating toluene on the sample at 500 rpm for 60 s. A waiting time of 5 min was observed between steps. Finally, the sample was encapsulated using a top 18 mm × 18 mm thin glass slide with double-sided tape at the edges to hold the top slide in place. Gaps between the bottom and top glass slides were sealed with epoxy and left to dry over 24 h in the N_2_ environment.

It must be noted that the optical characterization (PL, PLE, and TRPL) results presented in Figures 1–3 and 5 are based on the same monolayer in pristine and heterostructure form; that is, each measurement was performed before and after QD deposition.

### Optical Characterization

#### Steady-State Absorption and PL Spectroscopy

The absorption spectrum of the QDs was measured using a Shimadzu UV–vis spectrometer. A 0.1 mg/mL solution of colloidal QDs in toluene in a 1 cm cuvette was placed in an integrating sphere. A 1 cm cuvette filled with toluene was used as a reference. Steady-state QD PL in Figure 1c was obtained using a fluororemter (Edinburgh Instruments), with 0.1 mg mL^–1^ solution deposited in a 1 mm cuvette. Excitation wavelength was set to 500 nm, and PL was detected with an indium gallium arsenide array.

#### Steady-State Absorption Microscopy

The absorption spectrum of monolayer WS_2_ on the quartz substrate was measured with a Zeiss Axiovert inverted microscope in transmission using a halogen white light source *via* Zeiss EC Epiplan Apochromat 50× objective (numerical aperture (NA) = 0.95), forming a wide-field collection area diameter of 10 μm. Light transmitted *via* the sample was split with a beam splitter, with one component directed to a CCD camera (DCC3240C, Thorlabs) and the other coupled to a UV 600 nm optical fiber (200–800 nm spectral range) connected to a spectrometer (Avaspec-HS2048, Avantes).

#### Steady-State PL Microscopy

PL microscopy was performed using a Renishaw InVia confocal setup equipped with motorized piezo stage. Laser excitation was from an air-cooled Ar-ion 514.5 nm CW laser *via* a 50× objective (NA = 0.75). The sample was excited upside down to ensure that the monolayer was excited first *via* the thin substrate to avoid shadowing by the QDs once deposited. Signals were collected in reflection mode *via* a notch filter. The diffraction-limited beam spot size was estimated to be 0.84 μm. The PL signal was dispersed *via* a 600 l/mm grating prior to detection with inbuilt CCD detector. Laser power was measured directly *via* a 5× objective with a Thorlabs S130C photodiode and PM100D power meter.

The detection wavelength range for PL measurements was selected using the setup’s inbuilt WIRE software. The vis–NIR PL spectrum (Figure 1b) was generated with a 10 s integration at a single spot on the heterostructure. The corresponding QD PL spectrum was taken at a location away from the heterostructure. The NIR PL map (Figure 2a) was generated with 8 μm resolution and 2 s integration. The vis PL maps (Figure 5a) were generated with 2 μm resolution and 0.5 s integration. All PL measurements were performed at 0.44 μW (80.2 W/cm^2^).

Excitonic species were deconvoluted from pristine and heterostructure PL spectra using a procedure written in Matlab. The code incorporates the “gauss2” two Gaussian model fit. Further information on the Gaussian model is available *via* the *mathsworks* Web site.

#### Photoluminescence Excitation Microscopy

PLE measurements were performed using a custom-built inverted PL microscope setup. The inverted microscope arrangement enabled excitation of WS_2_ monolayer first *via* the thin glass slide, hence avoiding shadowing by the QDs. Variable wavelength excitation was provided by a pulsed super continuum white light source (Fianium Whitelase) *via* a Bentham TMc 300 monochromator. The optical image of the heterostructure was acquired using 600 nm laser light at low power *via* a 60× oil objective, producing a 200 μm circular wide-field image on an EMCCD camera (Photometrics QuantEM 512SC). A QD PL image of the heterostructure was obtained by filtering out the excitation wavelengths using a combination of 750 and 800 nm long-pass filters. Further precaution was taken to remove any long wave component in the excitation line using a 750 nm short-pass filter. An example of the QD PL image is given in SI Figure 6, which was recorded using 620 nm excitation at 10 MHz pulse rate (∼0.006 μJ/cm^2^ fluence) and 20 s integration time. The region of interest was isolated by closing an iris in the detection line just before the camera.

The procedure for obtaining PLE spectra is as follows: (i) The laser excitation *via* the monochromator was swept between the visible and NIR range. Given that the optics in the system were optimized for 600 nm and above, excitation was varied between 580 and 710 nm with 2 nm resolution. Each excitation was integrated for 20 s using 10 MHz pulses. (ii) The wide-field PL signal at each excitation was recorded, producing a spectrum of raw PL signal as a function of excitation wavelength. (iii) The background signal was obtained by covering the detector and repeating steps i and ii. The excitation power was recorded simultaneously using a Thorlabs S130C photodiode placed in the excitation line just before the sample, and a PM100D power meter interfaced with the data logging software. (iv) Raw data were postprocessed in *Origin*, where the background spectrum was subtracted from the raw PL spectrum and normalized by the number of photons injected at each wavelength. Finally, the PLE spectrum was corrected with a system calibration file based on the PLE and absorption spectra of a high PLQE NIR dye.

#### Time-Resolved PL Microscopy

TRPL measurements were performed using a PicoQuant Microtime 200 inverted confocal setup. Excitation was provided using 509 nm pulsed laser excitation *via* an inverted 20× air objective (NA = 0.4), with an estimated diffraction-limited spot size of 1.55 μm. Signals were detected with a single-photon avalanche diode.

For the WS_2_ monolayer PL decay, the repetition rate was set to 20 MHz with 25 ps resolution to obtain PL decay data. Laser excitation was filtered out with a 510 nm long-pass filter, and the NIR regions of both pristine and heterostructure PL were filtered out using a 700 nm short-pass filter, allowing for collection of WS_2_ PL only. All signals were scaled up to 1500 s, which was used on the lowest fluence measurement in the fluence series. Power was measured using an inbuilt photodetector at each fluence, which was previously calibrated in the same experimental conditions using a standard external power meter. Care was taken to ensure that measurements were made on the same spot on the monolayer before and after QD deposition. The instrument response function was measured with a blank glass cover slide as used for the sample. Decay rates were fitted using a model developed in Origin, which consists of a Gaussian (as the IRF) convoluted with a double exponential decay.

For PbS–CdS PL decay, excitation was provided using the 509 nm pulsed laser at 0.5 MHz repetition rate and 200 ps resolution. The excitation signal was filtered out using a 510 nm long-pass filter, and QD emission was isolated with an 800 nm long-pass filter, removing any signal from the underlying WS_2_.

#### Figure 1b Inset: Heterostructure Image

WS_2_ nanocrystal graphics were developed in VESTA software^54^ and parsed into ChemDraw3D (PerkinElmer) for rendering. QD graphics were modeled using Blender 3D modeling software.

## Supporting Information Available

The Supporting Information is available free of charge at https://pubs.acs.org/doi/10.1021/acsnano.0c05818. The data underlying all figures in the main text and supplementary information are available at the University of Cambridge data repository at https://doi.org/10.17863/CAM.58224.

Further information on PLE data normalization; details on photoluminescence contribution (PL_ctr_) derivation; PLE study on heterostructures with alternative quantum dot to two-dimensional material surface attachment ligands; detail on theoretical Förster resonance energy transfer radius estimation; optical image of region in which PL data in Figure 5a was obtained; energy transfer efficiency (eq 2) derivation and; wide-field quantum dot PL image on tungsten disulfide monolayer (PDF)
