# Supplementary material for: Directed Energy Transfer from Monolayer WS2 to Near-Infrared Emitting PbS–CdS Quantum Dots
Source: ACS Nano. 2020 Oct 20;14(11):15374–84. doi: 10.1021/acsnano.0c05818 (PMC8155326; doi:10.1021/acsnano.0c05818)
Supplement: Supplementary file 1 — nn0c05818_si_001.pdf [file nn0c05818_si_001.pdf]

Supporting Information

Directed Energy Transfer from Monolayer WS2 to near Infrared Emitting PbS-CdS Quantum Dots

Arelo O.A Tanoh[1,2], Nicolas Gauriot[1], Géraud Delport[1], James Xiao[1], Raj Pandya[1], Jooyoung Sung[1], Jesse Allardice[1], Zhaojun Li[1], Cyan A. Williams [2,3], Alan Baldwin[1], Samuel D. Stranks[1,4], Akshay Rao[1*]

[1]Cavendish Laboratory, Cambridge, JJ Thomson Avenue, CB3 0HE, Cambridge, United Kingdom

[2]Cambridge Graphene Centre, University of Cambridge, 9 JJ Thomson Avenue, Cambridge, CB3 0FA, Cambridge, United Kingdom

[3]Department of Chemistry, University of Cambridge, Lensfield Rd, CB2 1EW, Cambridge, United Kingdom

[4]Department of Chemical Engineering & Biotechnology Department of Chemical Engineering & Biotechnology, University of Cambridge, Philippa Fawcett Drive, Cambridge CB3 0AS, UK.

*E-mail: ar525@cam.ac.uk

Section 1: Photluminescence excitation (PLE) data scaling

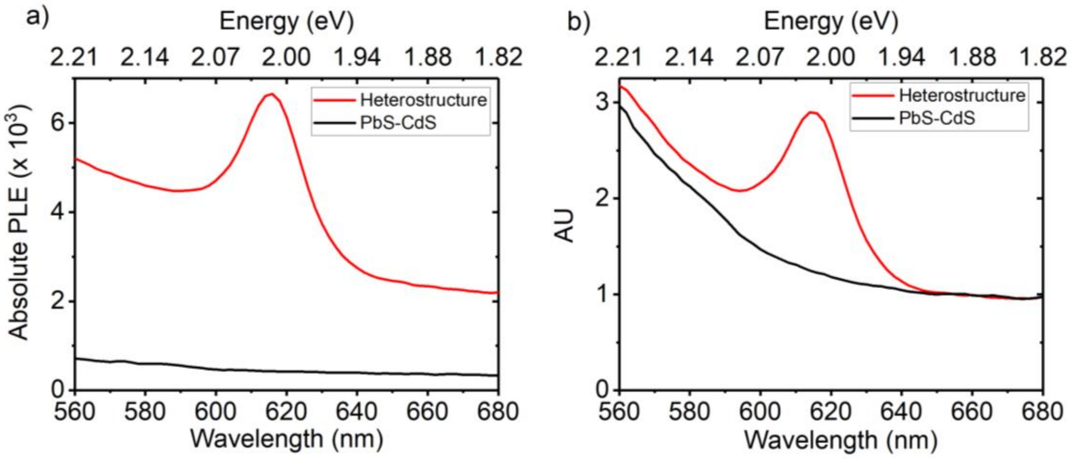

SI Figure 1 a-b: **a)** Unscaled heterostructure (red) and PbS-CdS (black) PLE data. **b)** Heterostructure and PbS-CdS PLE normalised to mean PLE values off-resonant to underlying WS_2 donor (670-700 nm).

Section 2: Photoluminecence contribution (*PL_{ctr}*) derivation

The photoluminescence contribution (PL_{ctr}) by the transition metal dichalcogenide (TMD) monolayer to the quantum dot (QD) emitter is derived. Vavilov's rule,[1] which states that PLQE is independent of excitation wavelength, forms the key assumption in this derivation. Given the range of wavelengths presented in PLE measurements (560 nm – 680 nm) this assumption is regarded as reasonable. We consider the photoluminescence excitation (PLE) of the QD at excitation resonant and non-resonant to the underlying WS_2 monolayer *i.e.* $PLE_{\lambda *}$ and PLE_{λ} respectively. In each case the PLE from the QD emission detection is given by *equations 1* and *2*:

$$PLE_{\lambda *} = \frac{PL_{\lambda *}}{n_{\lambda *}} = Abs_{\lambda *} \times PLQE \tag{1}$$

$$PLE_{\lambda} = \frac{PL_{\lambda}}{n_{\lambda}} = Abs_{\lambda} \times PLQE \tag{2}$$

Where *n* and *Abs* are the number of photons per second injected and absorption of the QDs. By dividing *equation 1* by *equation 2* we obtain:

$$\frac{PLE_{\lambda *}}{PLE_{\lambda}} = \left(\frac{n_{\lambda}}{n_{\lambda *}}\right)\frac{PL_{\lambda *}}{PL_{\lambda}} = \left(\frac{Abs_{\lambda *}}{Abs_{\lambda}}\right) \tag{3}$$

Hence the absorption ratio is equivalent to the ratio of WS_2 resonant PLE to non-resonant PLE. This is ratio is given by *R*:

$$R = \frac{PLE_{\lambda *}}{PLE_{\lambda}} = \left(\frac{n_{\lambda}}{n_{\lambda *}}\right)\frac{PL_{\lambda *}}{PL_{\lambda}} = \left(\frac{Abs_{\lambda *}}{Abs_{\lambda}}\right) \tag{4}$$

By comparing the *R* values on the heterostructure and the QD control, we can identify an additional contribution to the QD absorption *i.e.* ΔR from the underlying WS_2.

$$\Delta R = R_{Het} - R_{QD} \tag{5}$$

Expressing equation (6) as a proportion of the heterostructure R value (R_{Het}), we obtain the contribution of PL by the WS_2 to the QDs.

$$PL_{ctr} = \left(\frac{R_{Het} - R_{QD}}{R_{Het}}\right) = \left(\frac{\Delta R}{R_{Het}}\right) \tag{6}$$

Section 3: Corroborating Förster resonance energy transfer (FRET)

Section 3.1: PLE study on heterostructures with alternative quantum dot to two dimensional material (QD-2D) surface attachment ligands of increasing length

SI figure 2 shows the wide field PLE spectra of tungsten disulphide/ lead sulphide-cadmium sulphide (WS_2/PbS-CdS) heterostructures with varying QD-2D surface attachment thiol ligands. Table 1 lists the ligands used and their corresponding lengths. The difference in prominence of the WS_2 resonant peak is due to the variation in size of the WS_2 monolayers used. The 1,3 Benzene dithiol (BDT) sample has the largest monolayer and hence the most prominent WS_2 `A' exciton signal with less contribution of the QD emission shoulder blue of the $WS_2$ peak as seen in other samples. All signals were obtained by scanning about $WS_2$ `A' exciton and detecting and PbS-CdS emission (~ 900 nm). All signals show the WS_2 `A' exciton peak in the expected spectral region (614 – 620 nm).

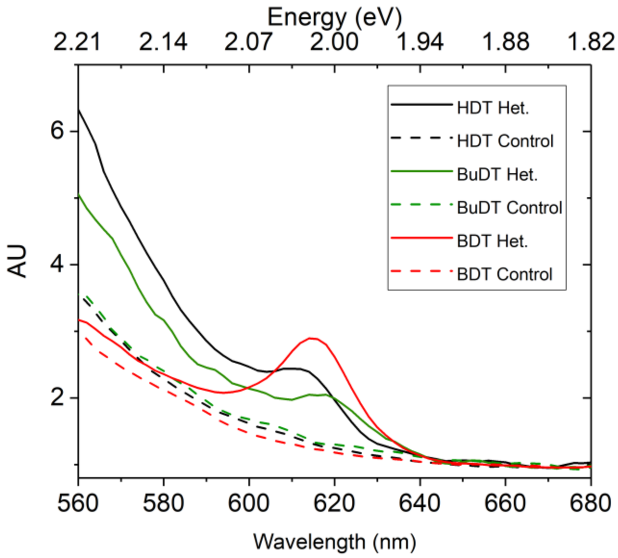

SI Fig 2: Normalised PLE spectra of 2D-QD heterostructures with varying surface ligands, namely BDT (red), Butane dithiol (BuDT) (green) and Hexane dithiol (HDT)(black). Dashed lines represent control signals.

Table 1: Dithiol ligands and corresponding lengths. *(*)* indicates value estimated using calculated dithiol ligand lengths from the open literature (Mispelon *et al.*,[2] Table 1, p. 18566) and the known bond angle of a hexagonal planar benzene ring

**Ligand**	**Length**	**Reference**
1,3 Benzene dithiol (BDT)	0.47 nm	*
1,4 Butane dithiol (BuDT)	0.68 nm	3
1,6 Hexane dithiol (HDT)	0.95 nm	3

While charge orbital overlap between donor and acceptor species is a possibility at these separation distances, the high oscillator strengths of the TMD donor and QD acceptor still render ET *via* dipole interaction (*i.e*. FRET) highly favourable,[4] and more significant than proximity dependent Dexter energy transfer (DET).

The Förster radius, R_0, is defined as the distance between donor and acceptor through which there is a 50% probability excitation transfer.[5] We therefore estimate the theoretical Förster radius to quantify the likelihood of FRET being the dominant ET mechanism from 2D → QD in the heterostructures measured.

Section 3.2: Theoretical FRET radius estimation

Considering the 2D TMD as an array of point-like emitters and the QD film as an array of point-like absorbers, the FRET radius, R_0, is defined in equation 7.[4] This system is also well approximated by a 2D quantum well donor and nanoparticle acceptors, which follows a d^{-6} distance dependence for non-radiative energy transfer.[6]

$$R_0^6 = \frac{9ln10}{128\pi^5 N_A}\frac{\kappa^2 PLQE_D}{n^4}J \quad (7)$$

N_A is Avogadro's number, n is the refractive index of the medium surrounding the FRET pair, $PLQE_D$ is the donor's intrinsic photoluminescence quantum efficiency and κ^2 is the dipole orientation factor, which is equal to 2/3 for randomly oriented dipoles.[7] J is the overlap integral between the area normalised emission spectrum,[4] F_D and acceptor absorption spectrum given by the acceptor molar extinction coefficient, ε_A.

$$J = \int_0^\infty F_D(\lambda)\varepsilon_A(\lambda)\lambda^4\, d\lambda \quad (8)$$

It must be noted that J is evaluated with the wavelength in [nm] and ε_A in [M^{-1} cm^{-1}]. To compute R_0, we must calculate the overlap integral J from measured $\varepsilon_A(\lambda)$ and $F_D(\lambda)$ data. The molar extinction coefficient is obtained *via* Beer Lambert's law (*equation 9*) for absorbance, A of a 0.1 mg ml^{-1} suspension of QDs in toluene of molar concentration c, measured with a 1 cm path length, l, cuvette.

$$A(\lambda) = \varepsilon_A(\lambda)cl \quad (9)$$

However, to obtain the molar extinction coefficient, the molar concentration, c of QDs in [M] is needed. The first step in calculating c involves estimating the QD size by solving *equation 10* provided by Moreels *et al.*[8] for PbS QDs of diameter D using their band gap energy, E_O. Since the QDs used consist mainly of a PbS core as per the modified preparation method originally developed by Neo *et al.*,[9] the use of equation 10 is considered reasonable. For the QDs used, where E_O ~1.76 eV, we get D ~ 2.4 nm.

$$E_0 = 0.41 + \frac{1}{0.0252D^2 + 0.283D} \quad (10)$$

We then calculate the QD volume assuming a spherical shape. This is followed by multiplying the volume by the density of PbS (7.6 g cm^{-3}) to obtain the mass of a single QD. Multiplying the mass of a single QD by the Avogadro number yields an estimate for the QD molar mass, Mr ~ 33128 g mol^{-1}. Dividing the known QD concentration of 0.1 g L^{-1} (ie 0.1 mg mL^{-1}) by the estimated QD molar mass Mr, yields c ~ 3.02×10^{-6} M. We rearrange *equation 8* for molar extinction coefficient in [M^{-1} cm^{-1}], which is shown in SI figure 3 along with area normalised donor emission, F_D.

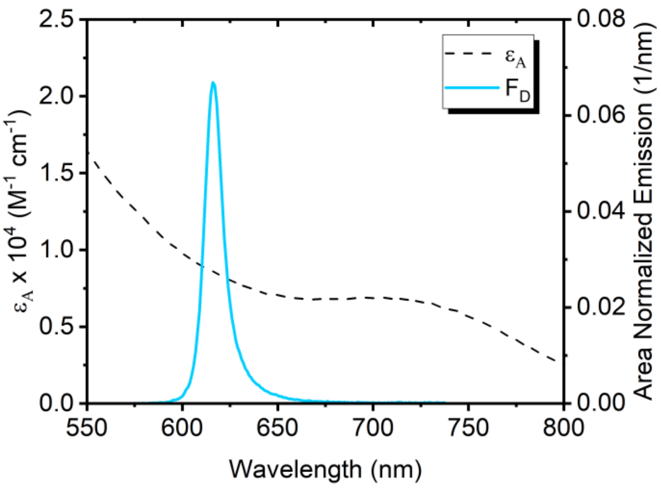

SI Fig 3: (Left axis) Molar extinction coefficient of 3.02×10^{-6} M PbS-CdS QDs in toluene measured with 1 cm cuvette. (Right axis) Area normalized WS_2 emission spectrum, F_D used to calculate overlap integral, J.

From the data shown in SI figure 3, the overlap integral is estimated *via equation 8* as $J \approx 1.23 \times 10^{15}$ M^{-1} cm^{-1} nm^4. Using a simplified version of *equation 7* below (*equation 11*) we estimate R_0 [nm] by assuming a vacuum between the emitter and absorber, *i.e.* n = 1 and orientation factor κ^2 = 2/3. For the ideal system, we assume the TMD donor to have unity PLQE. This approximation is however considered reasonable as we subsequently find that the energy transfer rate from WS_2 band edge to QD band edge outcompetes the intrinsic exciton quenching in WS_2, which is the known cause of low PLQE in newly prepared TMDs.

$$R_0 = 0.0211 \left(\frac{\kappa^2 PLQE_D}{n^4} J\right)^{\frac{1}{6}} \tag{11}$$

From *equation* 11, we obtain $\boldsymbol{R_0 \approx}$ **6.5 nm**, which exceeds the ligand separation distances between donor TMD and acceptor QD listed in table 1. This highlights the significance of the oscillator strength of the constituent heterostructure materials over their physical separation distance. This strongly implies FRET as the dominant ET process observed.

Section 4: WS_2 PL map region for PL scatter data shown in Figure 5.a

WS_2 PL scatter data presented in Figure 5.a were measured from the *64 µm x 48 µm* rectangular region (orange dashed lines) shown in the optical images of the monolayer below.

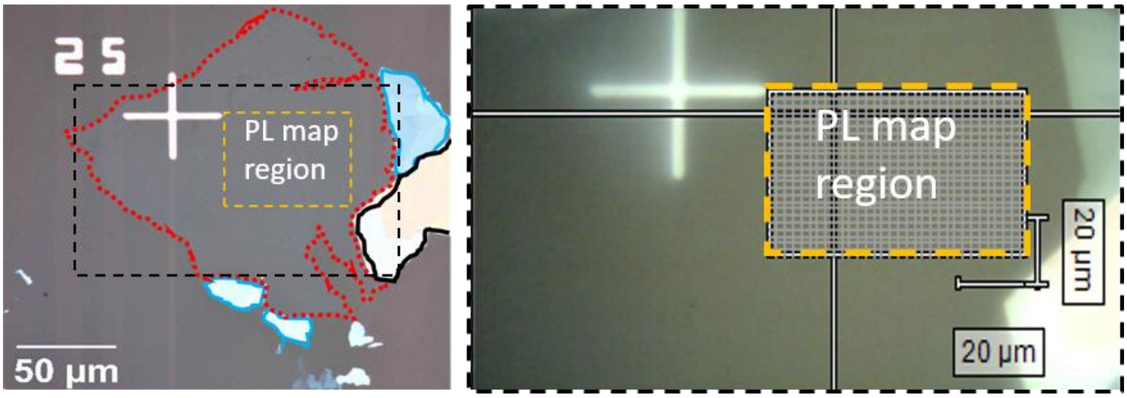

SI Fig 4:. LHS image shows monolayer region outlined in red dashed lines and estimated region of PL map is outlined in orange dashed lines. Black dashed region in LHS corresponds zoomed optical image on RHS taken with the Invia microscope described in the *experimental section* of the manuscript. Orange dashed lined in RHS represents actual 64 *µ*m × 48 *µ*m PL map region taken with invia microscope (see *experimental section)*. White mesh in RHS represents PL map resolution (2 *µm* × 2 *µm*) as described in *experimental section*.

Section 5: Energy Transfer efficiency derivation

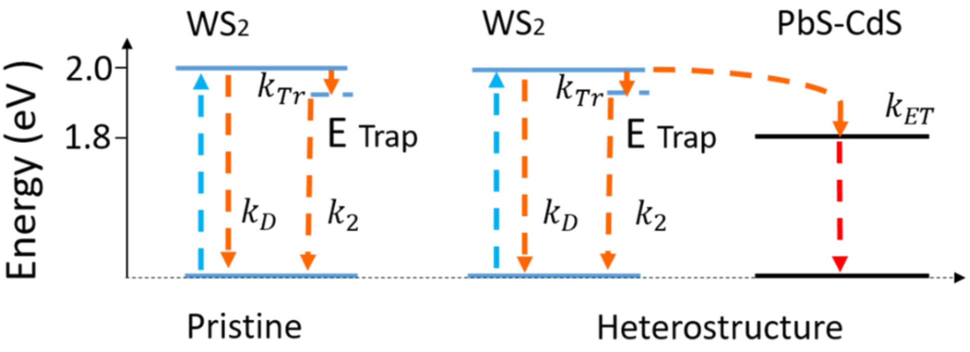

SI Fig 5: Energy level diagram illustrating radiative exciton pathways in pristine WS_2 (LHS) and in heterostructure. Blue arrows represent initial excitation, orange arrows represent WS_2 excitons and red arrows represent down-shifted excitons that recombine at lower energy in the PbS-CdS QD.

Table 2: Fitted PL lifetimes of pristine and heterostructure samples and resulting estimates for ET efficiencies. Fast components of WS_2 PL decay in heterostructure τ_1' and transfer efficiencies η_{ET} represent upper bound values due to limitations in instrument sensitivity.

Intensity	Pristine τ_1	Heterostructure τ_1'	Pristine τ_2	Heterostructure τ_2'
0.21 W cm^{-2}	0.456 ns	0.26 ns	3.63 ns	3.64 ns
63.4 W cm^{-2}	0.62 ns	0.26 ns	2.95 ns	2.9 ns

Following the RHS of SI Figure 5. WS_2 donor PL kinetics in the heterostructure can be described *via* the following set of related ordinary differential equations (ODEs):

$$\frac{dD^*}{dt} = -(k_D + k_{TR} + k_{ET})D^* \tag{12}$$

$$\frac{dTr}{dt} = k_{TR}D^* - k_2 Tr \tag{13}$$

Where D^* and Tr represent the WS_2 donor and trap state exciton populations respectively. The constants k_D, k_{TR}, k_{ET} and k_2 represent the donor's intrinsic recombination rate; intrinsic trapping rate; donor-acceptor energy transfer (ET) rate; and trap-ground state recombination rate respectively. By integration we arrive at the solutions to *equations 12* and *13*.

$$D^*(t) = D_0^* e^{-(k_D + k_{TR} + k_{ET})t} \tag{14}$$

$$Tr(t) = \frac{k_{TR}D_0^*}{[k_2 - (k_D + k_{TR} + k_{ET})]}\left(e^{-(k_D+k_{TR}+k_{ET})t} - e^{-k_2 t}\right) \tag{15}$$

Where D^*_0 represents the initial donor population. As such, the PL dynamics in the heterostructure can be defined as the sum of donor and trap population decay terms given by equations *14* and *15*:

$$PL(t) = D^*(t) + Tr(t) \tag{16}$$

i.e.

$$PL(t) = D_0^* e^{-(k_D+k_{TR}+k_{ET})t} + \left(\frac{k_{TR}D_0^*}{[k_2 - (k_D + k_{TR} + k_{ET})]}\left(e^{-(k_D+k_{TR}+k_{ET})t} - e^{-k_2 t}\right)\right) \tag{17}$$

In the absence of the QD acceptor the pristine WS_2 kinetics can be modelled by setting the transfer term $k_{ET} = 0$ so that:

$$PL(t) = D_0^* e^{-(k_D+k_{TR})t} + \left(\frac{k_{TR}D_0^*}{[k_2 - (k_D + k_{TR})]}\left(e^{-(k_D+k_{TR})t} - e^{-k_2 t}\right)\right) \tag{18}$$

The PL dynamics described by equations 17 and 18 consist of fast and slow decay components. In the pristine case (equation 18), at short time, *i.e.* t → 0, the fast decay time is given by:

$$\tau_1 \sim {}^{1}/_{(k_D + k_{TR})} \tag{19}$$

Similarly, in the heterostructure case (equation 17):

$$\tau_1' \sim {}^{1}/_{(k_D + k_{TR} + k_{ET})} \tag{20}$$

At long time *i.e.* t → ∞, given that the slow decay component (τ_2) remains unchanged for a given fluence (see Table 2), the slow decay time in both pristine and heterostructure cases is given as:

$$\tau_2 \sim \tau_2' \sim {}^{1}/_{(k_2)} \tag{21}$$

From equations 19 and 20, we can deduce the ET rate, k_{ET}, as:

$$k_{ET} = (k_D + k_{TR} + k_{ET}) - (k_D + k_{TR}) \tag{22}$$

The ET efficiency can then be determined in terms of rate constants. Using *equations 19, 20* and *22,* the ET can be simplified in terms of fast decay time constants as shown in *equation 2* of the main text:

$$\eta_{ET} = \frac{k_{ET}}{(k_D + k_{TR} + k_{ET})} = 1 - \frac{\tau_1'}{\tau_1} \quad (23)$$

Section 6: QD PL image on TMD monolayer

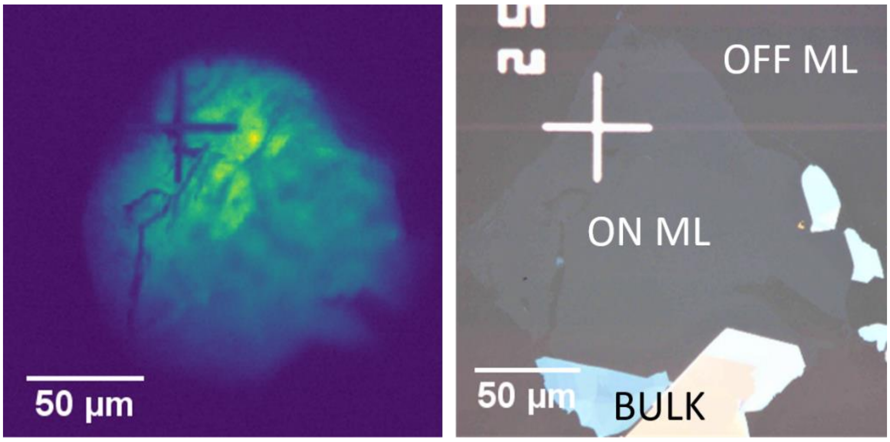

SI Fig 6: (LHS) Wide-field QD PL (900 nm) image of heterostructure at 620 nm excitation. (RHS) Optical image of monolayer used in heterostructure. QD PL clearly enhanced on TMD monolayer.

References

(1) Del Valle, J. C.; Catalán, J. Kasha's Rule: A Reappraisal. *Phys. Chem.* **2019**, *21*, 10061–10069. https://doi.org/10.1039/c9cp00739c.

(2) Mispelon, A.; Yan, J.; Milani, A. H.; Chen, M.; Wang, W.; O'Brien, P.; Saunders, B. R. Effects of Added Thiol Ligand Structure on Aggregation of Non-Aqueous ZnO Dispersions and Morphology of Spin-Coated Films. *RSC Adv.* **2015**, *5*, 18565–18577. https://doi.org/10.1039/c4ra15013a.

(3) Liu, Y.; Gibbs, M.; Puthussery, J.; Gaik, S.; Ihly, R.; Hillhouse, H. W.; Law, M. Dependence of Carrier Mobility on Nanocrystal Size and Ligand Length in PbSe Nanocrystal Solids. *Nano Lett.* **2010**, *10*, 1960–1969. https://doi.org/10.1021/nl101284k.

(4) Förster, T. Transfer Mechanisms of Electronic Excitation. *Discuss. Faraday Soc.* **1959**, *27*, 7–17.

(5) Guzelturk, B.; Demir, H. V. Near-Field Energy Transfer Using Nanoemitters For Optoelectronics. *Adv. Funct. Mater.* **2016**, *26*, 8158–8177. https://doi.org/10.1002/adfm.201603311.

(6) Hernández-Martínez, P. L.; Govorov, A. O.; Demir, H. V. Generalized Theory of Förster-Type Nonradiative Energy Transfer in Nanostructures with Mixed Dimensionality. *J. Phys. Chem. C* **2013**, *117*, 10203–10212. https://doi.org/10.1021/jp402242y.

(7) Melle, S.; Calderón, O. G.; Laurenti, M.; Mendez-Gonzalez, D.; Egatz-Gómez, A.; López-Cabarcos, E.; Cabrera-Granado, E.; Díaz, E.; Rubio-Retama, J. Förster Resonance Energy Transfer Distance Dependence from Upconverting Nanoparticles to Quantum Dots. *J. Phys.*

Chem. C **2018**, *122*, 18751–18758. https://doi.org/10.1021/acs.jpcc.8b04908.

(8) Moreels, I.; Lambert, K.; Smeets, D.; De Muynck, D.; Nollet, T.; Martins, J. C.; Vanhaecke, F.; Vantomme, A.; Delerue, C.; Allan, G.; Hens, Z. Size-Dependent Optical Properties of Colloidal PbS Quantum Dots. *ACS Nano* **2009**, *3*, 3023–3030. https://doi.org/10.1021/nn900863a.

(9) Neo, M. S.; Venkatram, N.; Li, G. S.; Chin, W. S.; Ji, W. Synthesis of PbS/CdS Core-Shell QDs and Their Nonlinear Optical Properties. *J. Phys. Chem. C* **2010**, *114*, 18037–18044. https://doi.org/10.1021/jp104311j.
